# Oxygen Saturation and Suck-Swallow-Breathe Coordination of Term Infants during Breastfeeding and Feeding from a Teat Releasing Milk Only with Vacuum

**DOI:** 10.1155/2012/130769

**Published:** 2012-05-09

**Authors:** Vanessa S. Sakalidis, Holly L. McClellan, Anna R. Hepworth, Jacqueline C. Kent, Ching Tat Lai, Peter E. Hartmann, Donna T. Geddes

**Affiliations:** School of Chemistry and Biochemistry, The University of Western Australia, M310, 35 Stirling Highway, Crawley, WA 6009, Australia

## Abstract

*Background*. Vacuum is an important factor in milk removal from the breast, yet compression is the predominant component of milk removal from bottle teats. Since bottle-feeding infants have lower oxygen saturation, vacuum levels, and different suck-swallow-breathe (SSwB) coordination to breastfeeding infants, we hypothesised that when infants fed from a teat that required a vacuum threshold of −29 mmHg for milk removal, that oxygen saturation, heart rate, and suck-swallow-breathe (SSwB) patterns would be similar to those of breastfeeding. *Study Design*. Infants (*n* = 16) were monitored during one breastfeed and one feed from the experimental teat. Simultaneous recordings were made of oxygen saturation, heart rate, vacuum, tongue movement, respiration, and swallowing. *Results*. There were no differences in oxygen saturation and heart rate between the breast and the teat. Infants displayed fewer sucks and breaths per swallow during nutritive sucking (NS) compared to non-nutritive sucking (NNS). The number of sucks per breath was similar for NS and NNS although respiratory rates were slower during NS. These patterns did not differ between the breast and the teat. *Conclusion*. These results suggest that vacuum may be conducive to safe and coordinated milk removal by the infant during both breast and bottle-feeding.

## 1. Introduction

Infant's coordination of the suck-swallow-breathe (SSwB) reflex is integral to safe, efficient, and effective breastfeeding. Healthy term breastfeeding infants are able to simultaneously suck and breathe and to suck and swallow but must briefly stop breathing to swallow, all while maintaining high blood oxygenation [1]. In contrast, during bottle-feeding infants often exhibit lower oxygen saturation than breastfeeding, periods of desaturation, and alternating periods of sucking and breathing [2–5]. It is often assumed that SSwB coordination during breastfeeding is similar to that of bottle-feeding despite some teats having large venting holes, rapid milk flow and high compressibility, whereas on the breast there is variable milk flow and limited compressibility [2, 3, 5–7]. These differences suggest bottle teat design may influence the mechanism by which an infant removes milk [2–4]. 

The level of intraoral vacuum applied by the infant is important for removal of milk from the breast [8]. Geddes et al. [9] showed that when the infant's tongue was in apposition with the palate, infants held a vacuum on average at −64 mmHg (baseline vacuum), and when the tongue lowered, vacuum increased in strength on average to −145 mmHg (peak vacuum) and milk flowed into the intra-oral cavity. This demonstrated that milk was removed using vacuum rather than compression of the nipple. In contrast, some bottle-feeding studies have demonstrated that infants do not require vacuum to obtain milk [10], which supports the theory that compression of the nipple and/or positive pressure is instrumental in milk removal [11, 12]. During bottle-feeding, infants have shown longer suck bursts [13], disorganised swallowing patterns, and lower oxygen saturation compared to breastfeeding [5]. Thus, in spite of the importance of vacuum in milk removal, how vacuum influences oxygen saturation, heart rate, and SSwB patterns during breastfeeding and bottle-feeding is not well understood. 

We hypothesized that when using only vacuum to remove milk from a teat, infants would show safe and well-coordinated patterns similar to breastfeeding. Therefore, an experimental teat was designed to release milk only when the infant applied a vacuum and used a similar tongue movement to breastfeeding. Geddes et al. [14] have confirmed that breastfeeding infants were able to successfully remove milk from the experimental teat using only vacuum (as opposed to compression). To verify that vacuum enabled the infant to control milk removal in a safe and coordinated manner, we measured oxygen saturation, heart rate, and SSwB patterns simultaneously on the breast and experimental teat. 

## 2. Materials and Methods

### 2.1. Participants

Mother-infant dyads were recruited through the Child and Adolescent Community Health Service (Oceanic Area Health Service), Perth, WA and via email notification at The University of Western Australia. Infants were healthy, full term, and without feeding difficulties, oral abnormalities (such as ankyloglossia or cleft-lip/palate), or illness. Mothers were breastfeeding and occasionally feeding their infant expressed breast milk via a bottle. Mothers supplied written informed consent to participate in the study, and ethics approval was obtained from the Human Research Ethics Committee at The University of Western Australia.

### 2.2. Protocol

Participants completed two visits to the research laboratory at the Breastfeeding Centre of Western Australia, King Edward Memorial Hospital, Perth. Each infant was monitored for a breastfeed during one visit and a feed using the experimental teat during the other visit. Simultaneous recordings were made of intraoral vacuum, tongue movement, respiration, oxygen saturation, and heart rate for the entire feed using a customised computerized data collection system (LactaSearch, Medela AG, Baar, Switzerland). 

### 2.3. Feeding Assessments

#### 2.3.1. Suck-Swallow-Breathe, Oxygen Saturation, and Heart Rate Monitoring

Submental imaging of the infant's intra-oral cavity was used to determine both tongue action and milk flow during all monitored feeds using a Toshiba SSA-770A/80, Aplio 80 (Tokyo, Japan) ultrasound machine with an endocavity convex transducer (PVT-661VT) and Parker Ultrasonic Gel (Fairfield, NJ, USA). This method enables both a clear view of the nipple, tongue, hard palate, soft palate, and milk flow into the intra-oral cavity [9, 14, 15]. Intra-oral vacuum was measured using a small silicone tube (Supplemental Nursing System, Medela AG, Baar, Switzerland) filled with sterile water [9, 14, 16]. One end was placed alongside either the mother's nipple or the teat and extended 1-2 mm beyond the tip, and the other end was attached via a silicone tube (650 mm × 4 mm) and a three-way tap to a pressure transducer (SP854, Memscap, Bernin, France) with disposable clip-on dome (MLA844, AD Instruments, Castle Hill, Australia) [9, 14, 16].

Patterns of respiration and swallowing were measured using respiratory inductive plethysmography (RIP) (Respitrace QDC, SensorMedics, Yorba Linda, CA, USA) from two bands, one placed around the thorax at the level of the nipples and a second around the abdomen at the level of the umbilicus. The output displayed the thoracic trace, the abdominal trace, and the sum of thoracic and abdominal effort. Bands were secured using micropore tape and were connected to the Respitrace. RIP has been validated against ultrasound as a highly reliable method for identifying swallows during breastfeeding [17] and has been used successfully to compare respiratory changes for breast and bottle-feeding in term infants [3, 5]. With RIP and other methods used to detect swallowing, degradation of the signal can occur due to excessive movement of the infant. Alternative methods used for swallowing detection during feeding are invasive and therefore risk interfering with breastfeeding. Thermistors detect changes in nasal temperature, however poor positioning of the sensor, differences in sensors and ambient air temperature often degrade the resulting signals. Pharyngeal pressure monitoring via an intranasal catheter is both invasive and may interfere with respiration during breastfeeding [17, 18]. Taking into account the limitations of RIP, any unsettled feeding/infant movement that altered the signal was noted during recording. Oxygen saturation and heart rate were recorded using pulse oximetry (Radical/MasimoSET V4.1) with a paediatric sensor (LNOP YI Multisite) taped to the distal end of the infant's foot. Outputs from the ultrasound machine, pressure transducer, RIP, and pulse oximeter were synchronised by the Lactasearch and recorded using the software package DIAdem (version 11.1, National Instruments, TX, USA) with a custom designed program for offline data analysis. Milk intake was measured by test weighing infants on an electronic scale (BabyWeigh Scale, Medela AG, Baar, Switzerland; resolution 2 g, accuracy ±0.034%) before and after each feed. 

#### 2.3.2. Experimental Teat 

**Figure 1:**
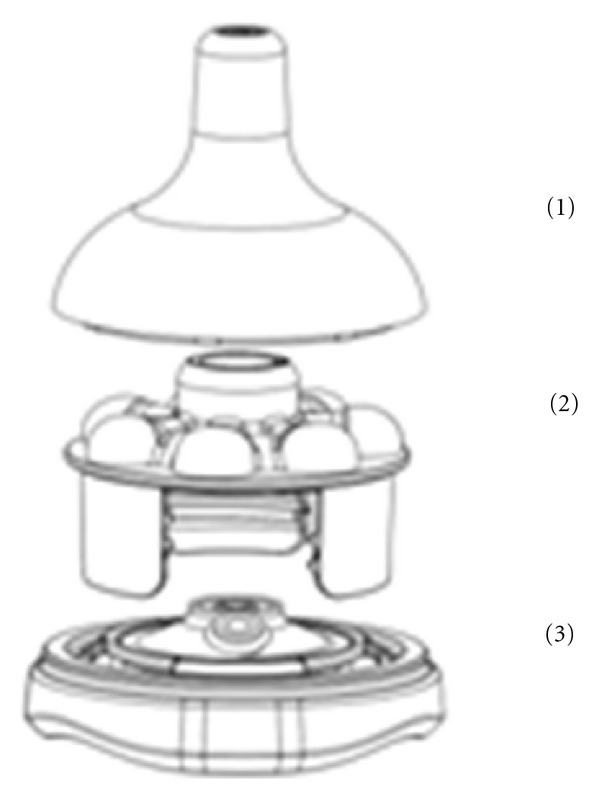
The experimental teat was comprised of 3 parts: (1) silicone teat, (2) middle teat base with raised support areas for the teat and a milk flow component, and (3) base contained a duck bill valve to vent the bottle.

The experimental teat was comprised of 3 parts: the hollow silicone top, the base, and a middle control component that regulated milk flow, depending on the level of vacuum applied by the infant and the size of the flow hole. At a threshold vacuum of −29 mmHg, the circular membrane deformed to allow milk flow through a channel at the side of the membrane. The infant was unable to remove milk using only compression of the teat by the jaw/tongue. The base included a duck-bill valve that vented the bottle (Figure 1). 

### 2.4. Data Analysis

#### 2.4.1. Suck, Swallow, and Breathe Variables

**Figure 2:**
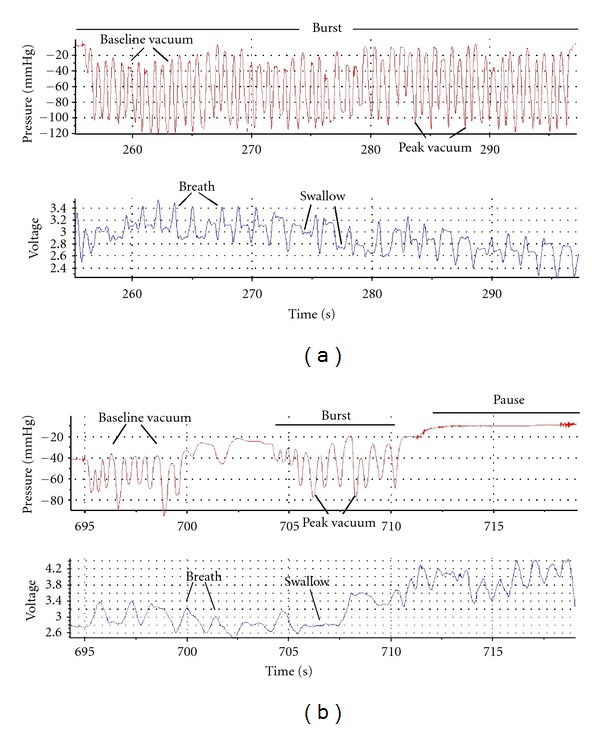
Trace of a suck-swallow-breathe burst of an infant during (a) nutritive sucking (NS) and (b) non-nutritive sucking (NNS) during the monitored breastfeed. During the NS burst, there are more sucks, swallows, and breaths, and the burst is longer than NNS.

**Figure 3:**
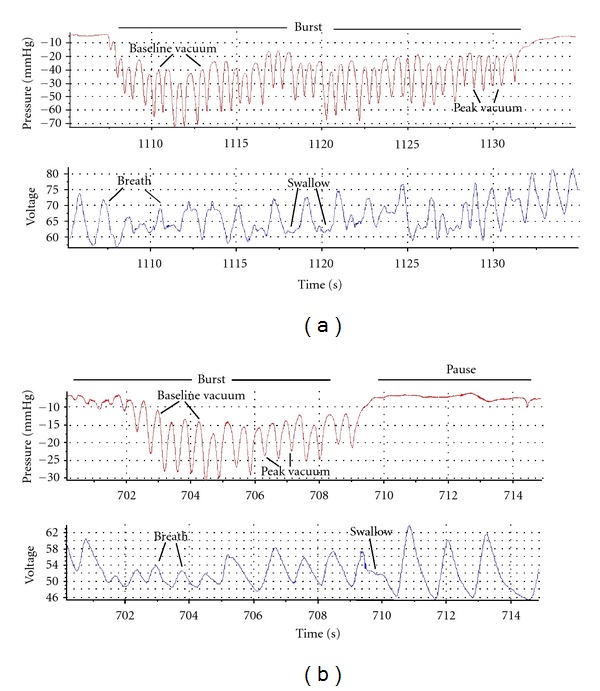
Trace of a suck-swallow-breathe burst of the infant during (a) nutritive sucking (NS) and (b) non-nutritive sucking (NNS) during the monitored feed from the teat. Similar to the breastfeed, during the NS burst, there are more sucks, swallows, and breaths and the burst is longer than NNS.

The customised script for DIAdem software (National Instruments) was used to extract intra-oral vacuum levels, respiration, swallow, oxygen saturation and heart rate measurements. Each feed was divided into suck bursts and pauses. Suck bursts were identified as the tongue moving on ultrasound and an active vacuum curve present and pauses as the tongue resting on ultrasound and a stable vacuum trace. Sucks were classified as nutritive sucking (NS) if milk flow was observed in the intra-oral cavity on ultrasound where the milk bolus appeared as a hypoechoic (black) area filled with echogenic white flecks (milk fat globules), nutritive pausing (NP) if the pause occurred directly after NS, non-nutritive sucking (NNS) if no milk flow was observed in the intra-oral cavity on ultrasound (Figures 2 and 3), and non-nutritive pausing (NNP) for subsequent pauses. Ultrasound has been used previously to identify milk flow (milk fat globules) during a suck cycle and suck burst [9, 14, 15]. A breath was defined by visualisation of both an inspiration detected as an increase in voltage, and expiration as a decrease in voltage. A swallow was identified as a stable signal on the trace [17].

For each NS and NNS burst across the entire feed on both the breast and teat, peak vacuum (mean minimum pressure, mmHg), baseline vacuum (mean maximum pressure; mmHg), mean vacuum (mmHg), suck rate (sucks/min), respiratory rate (breaths/min) and suck burst duration were calculated. For each pause across the entire feed, pause type (NP/NNP), mean vacuum, respiratory rate, and pause duration were calculated. Suck bursts and pauses were sequentially numbered to allow analysis of patterns across the feed. Nutritive transfer rates were calculated as the total milk transferred divided by the total duration of NS. Mean, minimum, and maximum oxygen saturation and heart rate were calculated for the entire feed. 

For the feeds on the breast/teat, the first three well-visualised NS and NNS bursts were selected and the number of sucks (S), swallows (Sw), and breaths (B) were counted. From this, SSwB ratios were determined by calculating the ratios of S : Sw and B : Sw relative to 1 swallow, and S : B relative to 1 breath. 

### 2.5. Statistical Analysis

Data analysis was performed using R 2.9.0 (The R Core Team) [19]. Packages nlme, [20], lattice [21], and multcomp [22] were used for linear mixed models, graphical exploration, and general linear hypothesis tests, respectively. Differences were considered significant when *P* < 0.05. Summary data is presented as mean ± SD or median (interquartile range). 

Feeding characteristics, oxygen saturation, and heart rate for the two feeds were compared using paired Student's *t*-tests following testing for normality using the Shapiro test or the Wilcoxon rank sum test otherwise. All other variables; the number of sucks, swallows, and breaths; SSwB ratios; sucking rates, respiratory rates; burst duration and burst vacuum levels, were compared using linear mixed models to account for the repeated measures in each feed. Models included individual intercepts as the random effect. Models were selected using forward stepwise regression using a *P* < 0.05 threshold. All nonsignificant predictors and interaction were omitted from the final models unless they were included in a higher level interaction. For all models, an interaction term for feed (breast/teat) type and burst type (NS/NNS/NP/NNP) or (NS/NNS) for sucking variables was included. To determine patterns across the feed for the variables suck rate; respiratory rate; mean/peak/baseline vacuum; burst order was considered as an additional interaction term. Relationships between mean, peak, and baseline vacuum with suck rate and respiratory rates were also tested by adding suck rate and respiratory rate as an additional interaction term. To determine the differences in vacuum level and burst duration between the feed and burst types, Tukey's multiple comparisons of means were made separately for each combination of vacuum level and burst duration for feed type (breast/teat) and burst (NS/NNS/NP/NNP) type. 

## 3. Results

### 3.1. Feed Characteristics

Eighteen mother/infant dyads were recruited. Two of the eighteen infants refused the teat, one of whom had a breastfeed recorded. Therefore, 16 infants with complete breastfeed and teat data were included in the analysis. At the first study session eleven infants were breastfed and six infants were fed using the teat. Infants were (mean ± sd) 49.4 ± 19.9 days old at the breastfeed and 56 ± 18.3 days old when they fed from the teat. Of the teat feeds, 14 were given by the mother, and the remainder were given by either a family member or a researcher. Milk intake during the monitored feed was significantly higher from the breast (*P* = 0.013); however, nutritive transfer rate (*P* = 0.59), feed duration (*P* = 0.25), and the duration of NS (*P* = 0.93) were not different between the breast and the teat (Table 1).

### 3.2. Oxygen Saturation and Heart Rate

No difference was seen in mean (*P* = 0.13), minimum (*P* = 0.81), and maximum (*P* = 0.33) oxygen saturation or mean (*P* = 0.56), minimum (*P* = 0.41), and maximum (*P* = 0.43) heart rate between feeds from the breast and the teat (Table 2). 

### 3.3. Number of Sucks, Swallows, and Breaths

During NS the number of breaths (*P* = 0.03) and swallows (*P* < 0.001) per burst was higher than the number during NNS. These patterns were similar between the breast and teat (breaths *P* = 0.7, swallows *P* = 0.38). The total number of sucks was higher during NS though the difference between NS and NNS was greater for the breastfeed (interaction; *P* = 0.04), (Table 3). 

### 3.4. Suck, Swallow, and Breathe Ratios

Fewer sucks per swallow were measured during NS than NNS (*P* < 0.001), with no difference in ratios between the breast and teat (*P* = 0.14). No difference was seen in the number of sucks per breath between NS and NNS (*P* = 0.08) or between breast and teat (*P* = 0.13). There were fewer breaths per swallow during NS, and an interaction by feed type (*P* = 0.001) showed, compared to the teat, when breastfeeding that the ratio was higher during NS but lower during NNS (Table 3). Mean SSwB ratios during NS were breast; 3.8 : 1 : 2.2 (range 1 : 1 : 1–12 : 1 : 4) and teat; 3.2 : 1 : 1.9, (range 1 : 1: 1–9 : 1 : 4) and during NNS were breast; 6.7 : 1 : 4.2 (range 2 : 0 : 1–23 : 4 : 23) and teat; 8 : 1 : 5.8 (range 2 : 0 : 2–15 : 1 : 8). 

### 3.5. Burst Characteristics

NS bursts on both the breast (Figure 2) and the teat (Figure 3) were significantly longer than NNS bursts, NP and NNP (all *P* < 0.001), and NS bursts were significantly longer at the breast than on the teat (*P* < 0.001). No differences in duration between the breast and teat were seen between NNS, NP, and NNP (all *P* > 0.05) (Table 4). Across the feed, an interaction (*P* = 0.0013) showed that NS bursts during breastfeeding became shorter as the feed progressed, but this pattern was not seen in teat feeds.

### 3.6. Sucking and Respiratory Rates 

Respiratory rate was not different between the breast and teat (*P* = 0.29). Respiratory rate during NS was slower than during NNS (*P* < 0.001), which was similar to NP (*P* = 0.55) and NNP (*P* = 0.81) rates. Sucking rates were significantly faster during NNS than NS (*P* < 0.001) and did not differ between the breast and teat (*P* = 0.61) (Table 5). Sucking (*P* = 0.34) and respiratory rates (*P* = 0.46) did not change across the feed.

### 3.7. Vacuum Relationships

Mean vacuum during NS (*P* < 0.05) was stronger than during NNS, which was stronger than NP (*P* < 0.001) and NNP (*P* < 0.001) for both breast and teat. All mean vacuum levels were different (all *P* < 0.05), with the exception of NP/NNP, which were similar within the breast (*p* = 0.85) and teat (*P* = 0.77). All mean vacuums were stronger at the breast (all *P* < 0.001) (Table 6).

Stronger peak vacuum was related to a slower suck rate for both the breast and teat, and this effect was greater for the teat (interaction; *P* = 0.021). No relationships were seen between suck rate and mean or baseline vacuum or between respiratory rate and mean, peak or baseline vacuum. Peak vacuum levels during NS were stronger at the beginning of the feed, and this effect was greater in the teat (interaction; *P* < 0.001). No difference was seen for baseline (*P* = 0.94) or mean vacuum (*P* = 0.93) across the feed. 

## 4. Discussion

This study has demonstrated that with the experimental teat, infants are able to maintain oxygen saturation and heart rates similar to those of breastfeeding if vacuum is made the central component of bottle-feeding. In addition, the experimental teat allowed infants to coordinate sucking, swallowing and breathing during both NS and NNS in a manner comparable to that used during breastfeeding. These results support recent evidence suggesting that intra-oral vacuum rather than compression is critical to ensure safe and coordinated milk removal from the breast [9]. 

### 4.1. Oxygen Saturation and Respiration

In contrast to many other studies [2–5, 23], oxygen saturation, heart rate, and respiratory rates were not different when infants fed from the breast or the experimental teat (Table 2). It is likely that infants were able to control the flow of milk more easily with the experimental teat than traditional teats as no milk would flow when they stopped sucking or if they compressed the teat; however, we have not measured other teats in this study. Traditional teats with high flow rates are associated with reduced oxygen saturation, altered respiratory rate and bradycardia in both term and preterm infants [2–5]. Certainly Goldfield et al. [5] found differences in oxygen saturation between breastfeeding and two different bottle systems, the first a soft-walled bottle and nipple, and the second a hard-walled bottle and nipple. The authors showed that oxygen saturation was significantly higher during breastfeeding than during feeds from the second bottle system only and that feeds from the first system showed higher values than feeds from the second system. Our results and those of others [5] suggest that the design of the bottle/teat influences infant oxygenation and show that the requirement of vacuum in the teat in our study enabled infants to be physiologically stable in a similar manner to the breast. These findings may be relevant to infants that are not physiologically stable when feeding from teats, such as premature infants where oral feeding with a teat often results in desaturation and bradycardic episodes when learning to feed [2, 4, 24, 25].

### 4.2. Suck, Swallow, and Breathe Coordination

Infant SSwB coordination was not compromised when feeding from the experimental teat. Differences between NS and NNS were similar for both breastfeeding and feeding using the teat. There was a greater difference in the total number of sucks between NS and NNS for the breastfeed compared to the teat (Table 3), which is most likely due to milk only being released from the breast during milk ejection, where milk flow rate increases and decreases rapidly over approximately 90 seconds. On average, there are 2.5 milk ejections in a breastfeed [26], and the infant must take advantage of these periods of increased milk availability in order to feed efficiently. In contrast, conventional teats often provide continuous milk flow and result in more sucks per burst compared to breastfeeding [13]. Certainly similar S : B ratios for the breast and experimental teat suggest that the infant was able to regulate its sucking and breathing such that it was able to maintain good oxygen saturation (Table 2) whether feeding from the breast or the teat. 

SSwB ratios were expected to differ between NS and NNS due to longer suck bursts and more frequent swallowing during milk flow, interestingly we showed this pattern on both the breast and teat. During NS the lower number of sucks per swallow (breast, NS; 3.1 versus NNS; 6.0, teat NS; 2.7 versus NNS; 6.5) and breaths per swallow (breast, NS; 1.9 versus NNS; 4.0, teat NS; 1.6 versus NNS; 5.0) on the breast and teat, was in agreement with Weber et al. [27] who noted that S : Sw ratios [28] increased from 1 : 1 to 2 : 1–3 : 1 later in the feed, suggesting a response to decreasing milk flow. Goldfield et al. [5] showed that during breastfeeding swallows occurred in an organised manner and did not appear to interrupt sucking, whereas during feeds from the second (hard-walled) bottle system, swallowing occurred more frequently and in a random/disorganised manner. The authors suggested these factors most likely contributed to the periods of desaturation occurring on the second system [5]. Again these results demonstrate that bottle design influences the infant's coordination and supports the notion that similar SSwB coordination between the breast and teat in this study is a result of the vacuum requirement for milk removal. It is not clear whether this is because the infant is regulating milk flow at will or that the coordination of tongue action and application of vacuum is also influencing SSwB coordination, but both are likely to be contributing factors. 

### 4.3. Burst Characteristics

Burst duration patterns were similar during breastfeeding and feeding from the experimental teat, though the longer NS bursts observed during breastfeeding may indicate that infants were maximising milk removal during milk ejection. As highlighted earlier, milk ejection is a transient phenomenon lasting approximately 90 seconds [29] and feeding is most efficient if the infant takes advantage of this period of increased milk availability, whereas with a bottle the milk is always available. Despite this, there was no difference in the total time spent NS between the breast and the teat (Table 1). The fact that pause durations were also similar between the breast and teat (Table 4) is consistent with the infants maintaining adequate oxygenation. Certainly preterm infants have shown longer pauses during bottle-feeding than breastfeeding indicating the need to recover from long sucks bursts [2]. 

The difference in milk transfer between the breast and teat may have been constrained by the volume of milk available in the bottle in that infants might have transferred a greater volume if more milk was available. Despite this, the NS transfer rate between the groups was the same. Contrasting results have been published with conventional teats. Taki et al. [13] showed that during a bottle-feed, despite similar milk transfer, suck bursts were longer and less frequent compared to breastfeeding. Our results show that the requirement of a threshold vacuum for milk removal enabled infants to regulate suck bursts and therefore allows for variation in infant feeding patterns across a feed, whereas other bottles that allow milk flow without sucking may interfere with the infant's ability to regulate suck bursts and alter patterns of breathing and swallowing [2–5]. 

### 4.4. Vacuum and Sucking Relationships

When feeding from both the breast and the experimental teat, NNS sucking rates were faster than that of NS, and mean vacuum levels were weaker during NNS, though vacuums were higher at the breast. Stronger breastfeeding vacuums can be explained by rapid changes in rates of milk flow (milk ejection) and the level of vacuum required for milk removal. During breastfeeding, the infant must apply a baseline vacuum to elongate and position the nipple within the oral cavity such that milk removal and swallowing is optimal [14]. The cyclic vacuum applied must then be strong enough to expand the nipple, and may be altered in response to changes in milk flow [14]. Both the threshold vacuum required for milk removal and flow rate of the teat are likely factors that have resulted in lower vacuums being applied by the infant during bottle feeding. Adaption to different feeding conditions has been shown previously, in particular to the rate of milk flow [30, 31]. Despite this, suck rates were not different for the breastfeed and feed from the experimental teat. Previously rates have been shown to differ with different types of teats, where higher flow teats are associated with faster suck rates and low flow teats with slower suck rates [31]. It is possible that the suck rate was similar between the breast and teat because infants were conditioned to sucking on the breast; however, given that previous studies have shown consistently higher suck rates on the bottle than the breast [13], we suspect that suck rates were similar for the vacuum release teat because the infant in fact controlled the rate of milk removal.

We have shown that by removing compression and relying on vacuum only for milk removal oxygen saturation, heart rate, and SSwB patterns were not different between the breast and teat. Previous studies have shown that infants apply an alternating pattern of compression and vacuum to traditional teats, where milk is removed when the tongue is compressing the teat producing positive pressure rather than as the tongue is lowering, creating negative pressure [10, 30, 32]. The experimental teat enabled some compression of the teat in the second half of the suck cycle when milk was cleared from the oral cavity but excluded the possibility of milk removal with compression in the first half of the suck cycle. Although infants were only exposed to the experimental teat once, they were able to produce SSwB patterns and vacuum in a similar manner to breastfeeding suggesting rapid adaptation [14]. The long-term outcomes of feeding from both the experimental teat and breastfeeding were not measured in this study and warrant further research. It is not clear whether the high acceptability of the teat was due to the central vacuum component or the intrinsic capability of the infant in its ability to adapt to different feeding environments, we suspect that both these factors may be important. 

## 5. Conclusion

This study has demonstrated that when the application of vacuum, rather than compression, is required for milk removal from a teat, oxygen saturation, heart rate, and SSwB patterns are not different to those measured during breastfeeding. These results suggest that vacuum may be conducive to safe and coordinated milk removal by the infant during both breast and bottle-feeding.

## Figures and Tables

**Table 1:** Feeding characteristics for the monitored breastfeed and the feed from the experimental teat.

Feeding characteristics	Breast	Teat	*P* value
Milk intake (g)	93 ± 36	62 ± 30	0.013
Feed duration (s)	626 ± 173	738 ± 336	0.25
NS duration (s)	263 (212, 373)	264 (128, 318)	0.93
Nutritive transfer rate (g/minute)	23.6 ± 14.8	20.3 ± 13.5	0.59

Results are mean ± SD or median (interquartile range).

NS: nutritive sucking.

**Table 2:** Oxygen saturation and heart rate for the monitored breastfeed and the feed from the experimental teat.

		Breast	Teat	*P* value
	Mean	98.6 ± 1.1	98.6 ± 1.2	0.13
Oxygen saturation (%)	Minimum	92.9 ± 4.9	94.9 ± 6.5	0.81
	Maximum	99.9 ± 0.3	99.9 ± 0.3	0.33

	Mean	160.7 ± 10.7	162.5 ± 12.3	0.56
Heart rate (bpm)	Minimum	139.0 ± 9.0	142.8 ± 13.1	0.41
	Maximum	178.2 ± 13.0	182.0 ± 18.2	0.43

Results are mean ± SD.

**Table 3:** Number of sucks, swallows, and breaths per burst and the suck: swallow: breathe (SSwB) ratios during the monitored breastfeed and the feed from the experimental teat during nutritive (NS) and non-nutritive (NNS) sucking.

	Burst	Breast	Teat	^ #^ *P* value
	NS	19.5 (12.0, 28.5)	10.5 (7.8, 15.3)	^ ii^0.04
Sucks	NNS	6.0 (5.0, 9.0)	6.0 (3.8, 9.8)	
	*P* value^∗^	^ ii^0.04		
	NS	6.0 (3.0, 11.3)	4.0 (2.0, 7.3)	0.38
Swallows	NNS	1.0 (0.0)	0.5 (0.0, 1.0)	
	*P* value^∗^	<0.001		
	NS	12.5 (7.0, 17.8)	7.0 (4.0, 10.3)	0.7
Breaths	NNS	4.0 (3.0, 6.0)	5.0 (2.8, 7.3)	
	*P* value^∗^	0.03		
	NS	3.1 (2.1, 4.9)	2.7 (1.7, 3.7)	0.14
Sucks per swallow	NNS	6.0 (4.0, 9.0)	6.5 (5.8, 10.0)	
	*P* value^∗^	<0.001		
	NS	1.7 (1.3, 2.0)	1.7 (1.2, 2.0)	0.13
Sucks per breath	NNS	1.5 (1.0, 2.0)	1.3 (1.0, 1.9)	
	*P* value^∗^	0.08		
	NS	1.9 (1.3, 2.5)	1.6 (1.3, 2.2)	^ ii^ *P* ≤ 0.001
Breaths per swallow	NNS	4.0 (3.0, 5.0)	5.0 (3.4, 8.3)	
	*P* value^∗^	^ ii^ *P* ≤ 0.001		

NS: nutritive sucking and NNS: non-nutritive sucking.

Results are median (interquartile range).

*P value between NS and NNS.

^#^ *P* value between the breast and teat.

^ii^Significant interaction between the breast and teat with NS and NNS.

**Table 4:** Duration characteristics for the monitored breastfeed and the feed from the experimental teat.

	Burst	Breast	Teat	^#^ *P* value
	NS	8.9 (4.5, 18.3)	5.9 (3.6, 11.7)	0.001
Sucking duration (s)	NNS	4.5 (3.1, 7.1)	2.7 (2.0, 4.9)	0.41
	*P value	0.001	0.001	

	NP	3.2 (1.9, 5.7)	2.8 (1.8, 4.5)	0.99
Pause duration (s)	*P value	0.001	0.001	
	NNP	2.9 (2.2, 4.1)	3.8 (2.2, 6.1)	1.0
	*P value	0.001	0.001	

NS: nutritive sucking, NNS: nonnutritive sucking, NP: nutritive pausing, and NNP: non-nutritive pausing.

Results are median (interquartile range).

*P value compared to NS.

^#^ *P* value between the breast and teat.

**Table 5:** Respiratory and sucking rate during the monitored breastfeed and the feed from the experimental teat.

	Burst type	Breast	Teat	^#^ *P* value
	NS	59.2 ± 21.5	55.1 ± 23.9	
	*P value	<0.001		
	NNS	68.1 ± 22.3	68.8 ± 19.5	0.29
Respiratory rate (breaths/min)	NP	68.7 ± 22.2	66.3 ± 31.8	
	*P value	0.55		
	NNP	70.2 ± 31.3	70.3 ± 31.0	
	*P value	0.81		

	NS	89.1 ± 18.8	88.4 ± 28.1	
Suck rate (sucks/min)	*P value	<0.001		0.61
	NNS	103.9 ± 21.2	105.7 ± 23.4	

NS: nutritive sucking, NNS: non-nutritive sucking, NP: nutritive pausing, and NNP: non-nutritive pausing.

Results are mean ± SD.

*P value compared to NNS.

^#^ *P* value between the breast and teat.

**Table 6:** Vacuum relationships during the monitored breastfeed and the feed from the experimental teat.

	Burst type	Breast	Teat	^#^ *P* value
	NS	−68.4(−92.3, −47.2)	−32.4(−40.5, −25.3)	<0.001
	*P value	0.02	<0.001	
	NNS	−52.9(−89.9, −31.8)	−21.4(−29.7, −15.5)	<0.001
Mean vacuum (mmHg)	NP	−15.3(−28.2, − 6.7)	−7.9(−11.3, − 4.7)	<0.001
	*P value	<0.001	<0.001	
	NNP	−14.0(−29.7, − 8.3)	−6.9(−8.0, − 5.4)	<0.001
	*P value	<0.001	<0.001	

NS: nutritive sucking, NNS: non-nutritive sucking, NP: nutritive pausing, and NNP: non-nutritive pausing.

Results are median (interquartile range).

*P value compared to NNS.

^#^ *P* value between the breast and teat.
